# Health literacy mediates the relationships of cognitive and physical functions with health-related quality of life in older adults

**DOI:** 10.3389/fpubh.2024.1355392

**Published:** 2024-03-14

**Authors:** Bik C. Chow, Jiao Jiao, Tuyen V. Duong, Holger Hassel, Timothy C. Y. Kwok, Minh H. Nguyen, Huaxuan Liu

**Affiliations:** ^1^Department of Sport, Physical Education and Health, Hong Kong Baptist University, Kowloon Tong, Hong Kong SAR, China; ^2^Dr. Stephen Hui Research Centre for Physical Recreation and Wellness, Hong Kong Baptist University, Kowloon Tong, Hong Kong SAR, China; ^3^School of Nutrition and Health Sciences, Taipei Medical University, Taipei, Taiwan; ^4^International PhD Program in Medicine, College of Medicine, Taipei Medical University, Taipei, Taiwan; ^5^Institute for Applied Health Sciences, Coburg University of Applied Sciences and Arts, Coburg, Germany; ^6^Berlin Institute for Health and Social Affairs, Berlin, Germany; ^7^Department of Medicine & Therapeutic and School of Public Health, The Chinese University of Hong Kong, Shatin, Hong Kong SAR, China; ^8^School of Preventive Medicine and Public Health, Hanoi Medical University, Hanoi, Vietnam; ^9^School of Physical Education and Sport Science, Fujian Normal University, Fuzhou, Fujian, China

**Keywords:** health literacy, older adults, cognitive function, physical function, health-related quality of life

## Abstract

**Background:**

Declining cognitive function (CF) and physical function (PF) relate to poorer health-related quality of life (HRQoL) in older adults. As health literacy (HL) facilitates health information utilization, it may mediate links between functionality and HRQoL appraisals. This study examined HL as an intermediary between joint CF and PF contributions and HRQoL in Hong Kong older adults.

**Methods:**

490 older adults aged 50–80 years completed assessments from March to July 2021. Health Literacy Survey Questionnaire Short Form 12 questions (HLS-SF12), Montreal Cognitive Assessment (MoCA), Senior Fitness Test (SFT) and 12-Item Short-Form Health Survey version 2 (SF-12v2) were used to assess HL, CF, PF and HRQoL, respectively. Path analysis tested a model with HL mediating CF/PF predictors and HRQoL outcome.

**Results:**

Results for direct effects indicated that CF significantly associated with PF (β = 0.115, SE = 0.012, *p* < 0.001), PF significantly connected to HL (β = 0.101, SE = 0.022, *p* < 0.001), and HL significantly related to HRQoL (β = 0.457, SE = 0.049, *p* < 0.001). Meanwhile, PF significantly linked to HRQoL directly (β = 0.156, SE = 0.025, *p* < 0.001) as well as indirectly (β = 0.046, 95% CI [0.028, 0.067]). Significant mediating effect of HL was found on the relationship of PF and HRQoL.

**Conclusion:**

Findings confirm CF and PF joint impacts on HL and HRQoL in older adults, elucidating HL’s mediating role in translating functionality levels into HRQoL. Fostering enduring health knowledge access may thus buffer effects of age-related declines on well-being. Results can inform interventions leveraging this pathway to promote resilient trajectories.

## Introduction

Health literacy (HL) is a contemporary topic in today’s healthcare research. It refers to “the personal, cognitive and social skills which determine the ability of individuals to gain access to, understand, and use information to promote and maintain good health” (1). As evidenced in the World Health Organization’s 2019 report on health promotion, strengthening HL across populations can empower citizens to take charge of their health needs (2). Studies indicate that nearly half (47%) of individuals in eight European countries have inadequate or problematic general HL (3). Inadequate HL is of particular concern for older adults, who often have increased demands for health information and services to maintain well-being. While global aging populations continue expanding, academic inquiry into how HL impacts quality of life for older adults remains insufficient, with most previous studies adopting a clinical orientation rather than a public health perspective (4–6). To promote healthy aging among older population in community settings, elucidating the role of HL vis-à-vis health status and quality of life is critical. The current study therefore sought to examine HL’s association with bodily functions and Health-related quality of life (HRQoL) in older adults, guided by the rationale delineated below.

### Relationships among HRQoL, CF, and PF, and the potential role of HL

Health-related quality of life (HRQoL) is a multidimensional concept used to assess how health conditions influence overall well-being and quality of life. It encompasses physical, psychological, and social well-being (7). Research shows that advanced age correlates with diminished HRQoL in older adults (8, 9). Declines in cognitive function (CF) and physical function (PF)—which can occur together or separately—are key health issues impacting HRQoL (10). CF includes memory, verbal fluency, letter and pattern comparison, listening span, and processing speed (10–13). Mild cognitive impairment is common in older adults (14, 15) and can cause difficulties conducting daily activities (16, 17). Lower cognitive levels may increase psychological problems like mental illness (18) or dementia (19). Meanwhile, PF is considered as the ability to perform activities of daily living (20). Age-related PF decline stems from decreasing muscle strength, bone deterioration, and organ function (10, 21), lowering mobility and independence, worsening quality of life (22). Adequate PF enables older adults to preserve functionality and independence in daily living without major difficulty or dependence on others (23). For older adults, preserving cognitive and physical health helps enable better quality of life (24).

As some older adults experience age-related changes in cognitive and functional status, having adequate health literacy (HL) skills enables self-management of health and preservation of independence for older adults. Yet little research has examined the interconnections between cognitive function (CF), physical function (PF), HL, and health-related quality of life (HRQoL) concurrently (25, 26). Older people with more adequate CF and PF would suffer less physical or mental difficulties and be more likely to perceive themselves as better able to achieve daily activities, indicating higher perceived behavioral control (25), which closely relates to HL (27, 28). HL represents abilities to address health issues, and positively impacts HRQoL (4, 29). A framework suggests individual traits (including CF and PF) directly impact HL, which subsequently influences health outcomes (26). Accordingly, positive outcomes could enhance functional independence and quality of life (1). HL has long been considered a mediating factor due to its potentially modifiable influence on personal/social determinants of health (30). Therefore, it is reasonable to hypothesize HL as a viable mediator between CF and PF jointly and HRQoL. Elucidating HL’s ability to potentially buffer, mitigate or mediate these relationships allows for targeted improvements in older people’s healthcare policy, planning and education to promote healthy aging.

### The impact of HL on CF, PF, and HRQoL

Health literacy (HL) significantly impacts health management activities and outcomes (31–33). Inadequate HL often causes difficulty understanding and implementing health information, hindering health management (6). Older people face higher risk for low HL than younger populations (11, 34). Outcomes of limited HL include self-care problems like taking medications, managing chronic disease, utilizing preventive services (35, 36), and poorer health (37), negatively impacting health-related quality of life (HRQoL). Although prior studies suggest a strong HL-HRQoL linkage, findings vary across countries and cultures (38). Some research finds associations between low HL and poorer HRQoL (39–41), while other studies find no relationship (42). Further investigation on HL’s impact on HRQoL in aging populations is warranted.

As for the relationship between health literacy (HL) and cognitive function (CF) and physical function (PF) jointly, previous research has shown significant connections with poor HL separately. First, lower HL associates with worse CF (11–13). Some research also indicates CF highly impacts constructing HL in older adults (34, 43). Moreover, age-related CF decline may lead the older adults to feel shame and embarrassment, diminishing effective communication and exacerbating HL issues (44). Second, inadequate HL associates with limited PF (31, 35, 45–47). While most literature shows HL impacts PF, evidence also suggests PF may reciprocally influence HL, as differing PF levels produce different situational needs that fluctuate HL (30, 46, 47). Understanding these relationship details is important for mitigating CF and PF impacts on health-related quality of life (HRQoL). However, little research has examined HL, CF and PF interconnections or their combined influence on older adults’ HRQoL.

### The CF and PF interplay

Although cognitive function (CF) and physical function (PF) often concurrently decline in older adults, their interdependency remains unclear with inconsistent findings. A few studies demonstrate CF and PF associations (48, 49) but rarely explore predictor relationships. PF requires motor coordination and balance, which utilize cognitive skills like coordination and information processing, suggesting CF could affect PF (50). Meanwhile, some interventions using aerobic exercises (e.g., Yoga, Tai-chi) effectively promote CF (51, 52), indicating PF impacts CF. However, other research found non-significant CF-PF links after controlling for age, claiming their effects may not be fully distinguishable (53). Overall, limited knowledge exists regarding the CF and PF interplay.

### The current research

While prior research suggests socioeconomic factors influence health literacy (HL) and health-related quality of life (HRQoL) connections (30), most studies predominantly focus on these social factors, rarely considering how declining bodily functions, particularly cognitive function (CF) and physical function (PF), impact this relationship. Functional differences may lead to varying situational demands and complexities. Underestimating functional determinant effects or confusing HL’s potential role can cause inappropriate health outcome explanations (54). This study tested HL’s mediating role between combined CF and PF and HRQoL in older adults in Hong Kong. Clarifying HL’s role and revealing its potential effects provides evidence to promote well-being and HRQoL in older adults. Moreover, this research utilized lab observation for rigorous PF data collection from a relatively large sample to support the findings. Results may inform future empirical research and interventions to improve HL and HRQoL among older populations. Health services targeting low PF or CF can be tailored and practical strategies to reduce disparities considered based on this study.

### Hypothesis

**Figure 1:**
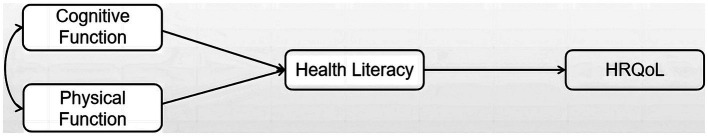
General theoretical model relating cognitive function, physical function, health literacy status to health-related quality of life outcomes.

A theoretical model was developed to examine the synergistic relationship between cognitive function (CF) and physical function (PF), and how they jointly affect health-related quality of life (HRQoL) through the mediator of health literacy (HL). Specifically, the model proposes that CF and PF combine to contribute directly to varying levels of HL. Meanwhile, HL may act as an intermediary between the joint contribution of CF and PF, and eventual HRQoL. In other words, the interaction of CF and PF is hypothesized to firstly impact HL levels, which in turn affects HRQoL. Therefore, the relationship between CF and PF is expected to contribute indirectly to HRQoL, by first influencing HL levels, which then lead to HRQoL. The proposed structural model illustrating these pathways is shown in Figure 1. The model suggests the following hypotheses:

*Hypothesis 1:* CF and PF levels combine to directly impact HL.

*Hypothesis 2:* HL mediates the relationship between CF and HRQoL.

*Hypothesis 3:* HL mediates the relationship between PF and HRQoL.

*Hypothesis 4:* Increased HL levels contribute positively to HRQoL.

## Methods

### Participants

Chinese adults ages 50–80 residing in the community were recruited to participate through social media, posters and flyers. Exclusion criteria aimed to ensure a relatively homogeneous sample appropriate for assessing culture-specific HL correlated with health status (55). Subjects were ineligible if they had severe cognitive/hearing impairment, inability to walk independently, lack of Chinese language proficiency, or less than 10 years resided in the city. Inclusion criteria were: (1) community-dwelling adults aged 50–80 years; and (2) ability and consent to participate in a one-hour face-to-face assessment session including health literacy (HL), cognitive function (CF), health-related quality of life (HRQoL) by questionnaires, and physical function (PF) by a series of physical fitness tests. Through direct communication and observation, the research assistant first inquired with potential participants about their language proficiency and hearing ability, then determined whether participants could respond clearly during a normal conversation. After this screening procedure, individuals who met the language and hearing requirements were invited to participate in the study. A total of 500 participants completed questionnaires and physical fitness tests conducted in an indoor laboratory. After data cleaning for missing information, the final sample was comprised of 490 older adults [female: 355 (72%); male: 135 (28%)] and their mean age was 66.04 ± 0.28 years old. For this sample, half of the participants (54%) were within 60–69 years old, while 15 and 31% were of age range in 50–59 and 70–80, respectively. Strict eligibility criteria ensured recruitment of an appropriate sample to address the study aims of examining correlations between culture-specific health literacy and health indicators among older Chinese adults.

### Ethical considerations

All participants were informed of the objectives of the study, the procedure of data collection, and the potential risks of physical tests. Prior to their participation, each participant had signed a written consent. The consent form stated that all data were anonymous, encrypted and stored safely with limited access to the principal investigator only. To ensure privacy and confidentiality, all personal information would not appear publicly in any form. Data and the coded list would be destroyed a year upon completion of the study. This study obtained ethical approval from the Research Committee of the University (Ref. No.: REC/19-20/0162) and was conducted in accordance with the established ethical guidelines.

### Procedures

Data collection was conducted between March to July, 2021. Participants signed informed consent before taking the survey. During the survey, participants completed self-reported questionnaires included basic demographics, health literacy (HL), cognitive function (CF), health-related quality of life (HRQoL) measures. Also, they took a battery of physical fitness tests to measure physical function (PF), including muscular strength, endurance, balance, agility and flexibility.

### Measurements

#### Independent variable

##### Cognitive function

CF was assessed by the Montreal Cognitive Assessment (MoCA) (56). The MoCA is used widely for testing CF among the older people, the Hong Kong version of MoCA has been validated (Cronbach’s α = 0.767) (57). It consists of 30 test items for assessing six areas of cognitive domains, including short-term memory, visuospatial abilities, executive functioning, phonemic fluency, verbal abstraction, attention, concentration, and working memory, language, repetition of complex sentences, and orientation to time and place. The total score of the MoCA ranges from 0 to 30, with a score of 26 and higher considered as normal and 26 or lower as an indicative of cognitive impairment.

##### Physical function

PF was assessed by the Senior Fitness Test (SFT) (58). It is widely used to evaluate physical function in the older people who are healthy with or without cognitive impairment for both research and clinical purposes. The SFT is a six-item physical fitness battery that includes Chair Stand, Biceps Curl, Chair Sit-and-Reach, Back Scratch, 2.4-m Up-and-Go, and 2-min Step in Place tests. Test scores can be compared to the normative values obtained from a sample of healthy Hong Kong older people (59). Scores of the six unweighted subscales will be transformed into percentile scores from 0 to 100 by gender and the average of these scores is the final score of PF.

#### Dependent variable

##### Health-related quality of life

HRQoL was assessed using the 12-item Short-Form Health Survey Questionnaire (SF-12v2) (60), which is the most widely used generic HRQoL instrument for evaluating functional health and well-being. The SF-12v2 was originally adapted from the Medical Outcomes Study 36-item Short-Form Health Survey (SF-36) (61) to retain its eight domains: Bodily Pain, General Health, Vitality, Social Functioning (each with one item); and Physical Functioning, Mental Health, Role Physical, and Role Emotional (each with two items). Notably, the “Physical Functioning” domain asks about perception of limitation in moderate physical activities and stair climbing, to determine the extent to which these activities affect daily life. It differs from the “Physical Function (PF)” measured by the SFT in the current research because the SFT is a physiological measure involving physical movements, a strict index describing people’s muscular performance and bodily abilities. The abbreviated SF-12v2 enables assessment even among those with limited attention spans or mental health problems. Following the procedures used in Lam et al. (60), the Physical and Mental summary scale scores are derived first by z score transformation and then summation of the scale scores is transformed into the population mean of 50 and standard deviation of 10. The range of HRQOL total score is from 0 to 100 with out of range scores set to either the high or low limit accordingly. The Chinese version has been validated (Cronbach’s α = 0.67–0.82 for different subscales) (60).

#### Mediate variable

##### Health literacy

HL was assessed by the 12-item Short-Form (HLS-SF12) of the 47-item European Health Literacy Survey Questionnaire (HLS-EU-Q47) (62). The HLS-SF12 is composed of 12 4-point Likert Scale items, which ranged from very difficult to very easy, and it retains the original 12 hypothetical components of comprehensive HL demonstrated in the HLS-EU-Q47. The HL matrix is constructed from four steps of information processing (finding health information, understanding health information, judging health information, and applying health information) interacting with three health domains (health care, disease prevention, and health promotion), which created a total of 12 dimensions or components of HL (63). The sum of the scores is the total score of HL. Total score computation follows the procedures used in Duong et al. (62), and the current study transformed the score into a 0–100 scale instead. The HLS-SF12 has been validated across six Asian countries (62) as being valid and reliable (Cronbach’s α = 0.85).

### Data analysis

This study proposed a theoretical model where cognitive function (CF) and physical function (PF) have a synergistic relationship, which is associated with varying health literacy (HL) status and subsequent impacts on health-related quality of life (HRQoL). The analysis was conducted in three stages. First, descriptive statistics characterized the variables. Next, Pearson correlations examined associations between HL, CF, PF, and HRQoL measures (HLS-SF12, MoCA, SFT and HRQoL), with age adjusted as a covariant. Finally, path analysis tested the overall theoretical model, including relationships among the multiple predictor and outcomes. Path analysis enables simultaneous testing of models with multiple dependent variables, making it well-suited for this study’s proposed model. In summary, the descriptive, correlation, and path analyses were conducted sequentially to provide insights into the relationships in the hypothesized model.

Descriptive statistics and bivariate correlations for all variables were calculated using IBM SPSS Statistics version 25.0 (IBM Corp., Armonk, N.Y., United States). Cases with missing data were handled by listwise deletion. Means, standard deviations and further statistical analyses included Pearson correlation were examined.

The proposed theoretical model was tested using structural equation modeling (SEM) software, Mplus 8 (64). Path analysis examined how health literacy (HL) status could relate to cognitive function (CF) and physical function (PF) and concurrently act as a mediator between health-related quality of life (HRQoL) and CF and PF. The regression coefficient (β) and standardized regression coefficient (*β) showed the effect size of relationships in the model, while *β was obtained using z-scores for regression instead of raw scores. With a sample size of 500, there was adequate power for SEM testing (65, 66).

The model was tested using maximum likelihood parameter estimation (MLPE) with listwise deletion of missing cases. Model fit was evaluated using: chi-square goodness-of-fit (χ^2^), comparative fit index (GFI), Tucker-Lewis index (TLI), standardized root mean residual (SRMR), and root mean square error of approximation (RMSEA) (66). Good fit was defined as: non-significant χ^2^; CFI and TLI > 0.95, SRMR and RMSEA <0.05. Bootstrapping with 5,000 samples was utilized to examine the indirect effects (67).

## Results

A summary of participants’ demographic information is presented in Table 1, showing the descriptive statistics for health literacy (HL) (Mean = 3.28, SD = 0.50), cognitive function (CF) (Mean = 26.3, SD = 3.28), physical function (PF) (six items’ Means and SDs, see Table 1), and health-related quality of life (HRQoL) (Mean = 3.87, SD = 0.61). Table 2 shows the partial correlations among variables as controlled by age variation. The results indicated all partial correlations were significant with the exception of the correlation between CF and HRQoL (*r* = 0.06, *p* = 0.226). However, their relationship was significant if not adjusted by age variance (*r* = 0.09, *p* < 0.05).

**Table 1:** Descriptive statistics of whole sample and by gender of health literacy, cognitive, physical functions and health-related quality of life measures.

Variable	Whole sample (*N* = 490)	Female (*N* = 355)	Male (*N* = 135)
	Mean, SD	Mean, SD	Mean, SD
Age	66.04, 6.26	65.93, 6.32	66.38, 6.11
HL	3.28, 0.50	3.26, 0.52	3.31, 0.44
CF	26.30, 3.28	26.24, 3.36	26.47, 3.08
PF (Fit-Chair Stand, counts)	15.13, 5.17	14.82, 5.01	15.94, 5.53
PF (Fit-Arm Curl, counts)	16.04, 4.82	15.54, 4.48	17.33, 5.43
PF (Fit-Step Test, counts)	91.55, 19.99	89.95, 19.04	95.75, 21.81
PF (Fit-Up Go, s)	5.00, 1.43	5.10, 1.44	4.74, 1.38
PF (Fit-Leg Extend, cm)	8.22, 12.86	10.16, 12.07	3.09, 13.52
PF (Fit-Back Scratch, cm)	1.41, 7.78	2.52, 6.87	−1.52, 9.18
HRQoL	3.87, 0.61	3.80, 0.62	4.03, 0.53

**Table 2:** Pearson correlations of all variables controlled by age variation (*N* = 490).

	HL	CF	PF	HRQoL
HL				
*r*	1	0.13	0.19	0.42
*p* value	—^a^	0.006	<0.000	<0.000
CF				
*r*	—	1	0.28	0.06
*p* value	—	—^a^	<0.000	0.226
PF				
*r*	—	—	1	0.3
*p* value	—	—	—^a^	<0.000
HRQoL				
*r*	—	—	—	1
*p* value	—	—	—	—^a^

a Not available.

**Figure 2:**
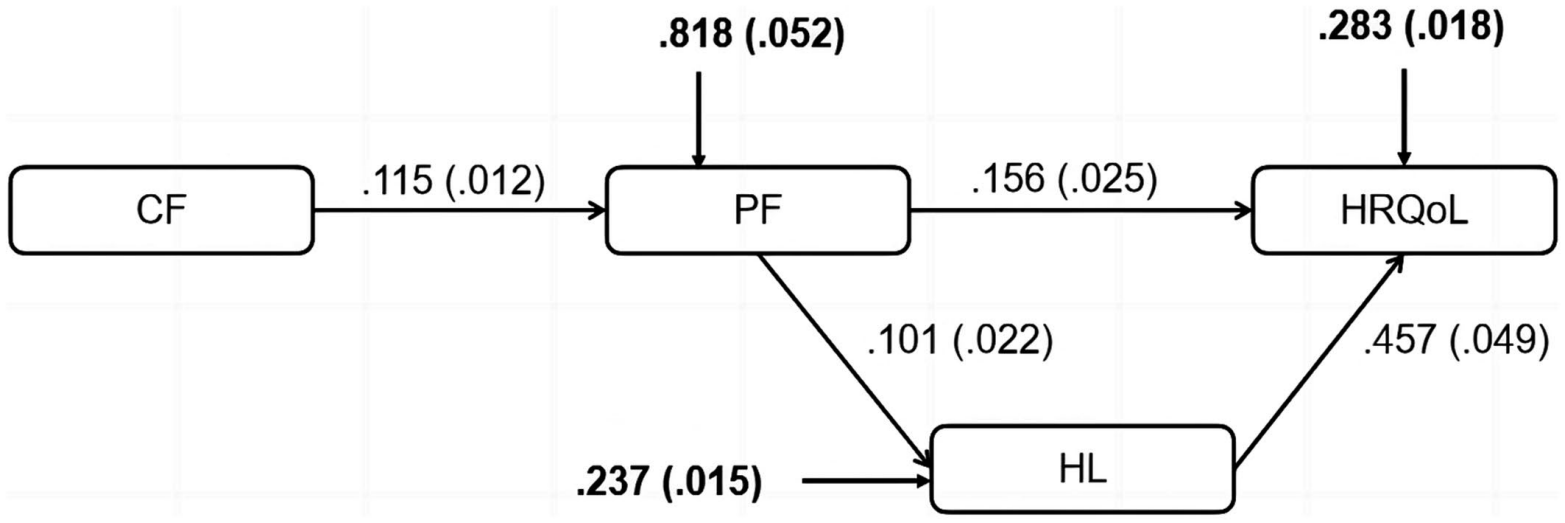
Model testing of direct and indirect effects.

To investigate whether health literacy (HL) would mediate the relation between cognitive function (CF) and physical function (PF) towards health-related quality of life (HRQoL), a path model was tested using Mplus Version 8 (64). An adequate good-to-fit model was indicated with the fit indices as χ^2^ = 4.759, χ^2^/df = 2.380 (*p* = 0.0926), CFI = 0.988, TLI = 0.964, RMSEA = 0.053, SRMR = 0.019. Figure 2 shows the significant direct and indirect effects of the variables involved in the model. Results for direct effects indicated that CF significantly predicted PF, β = 0.115, SE = 0.012, β* = 0.385, *p* < 0.001; PF significantly predicted HL, β = 0.101, SE = 0.022, β* = 0.200, *p* < 0.001; and HL significantly predicted HRQoL, β = 0.457, SE = 0.049, β* = 0.373, *p* < 0.001. Meanwhile, PF significantly predicted HRQoL directly, β = 0.156, SE = 0.025, β* = 0.242, *p* < 0.001. As would be expected from these results, the indirect effect from PF to HRQoL, tested using bootstrapped standard errors, was also significant, β = 0.046, 95% CI [0.028, 0.067], β* = 0.075. These findings partially support the hypothesized mediational model.

For the computation of mediating effect, the standardized betas were used. The direct effect of physical function (PF), after taking into account the mediation effect of health literacy (HL), on health-related quality of life (HRQoL) is β1* = 0.242 and the total effect of PF on HRQoL without the mediation by HL is β0* = 0.321. Thus the mediating effect of HL is determined by subtracting the direct effect from the total effect, i.e., β0* − β1* = 0.079. Therefore, the proportion of mediating effect to the total effect of influence in the model is 0.079/0.321, which is 24.6%.

The results reported above show that health literacy (HL) mediates the relationship between physical function (PF) and health-related quality of life (HRQoL). With these results, it would be interesting to see whether additional independent variables, such as Age, can act as a moderator that could influence the mediating effect between PF and HRQoL. In order to investigate the moderation effect of age on the relationship between PF and HRQoL, an interaction term, i.e., Age*PF, was added into the hypothesized model. The results are reported in Table 3. The results show that HL mediates the relationship between PF and HRQoL and Age*PF is not significant (β = 0.000, SE = 0.001, *p* = 0.943). This new analysis is consistent with the findings from the previous results. A comparison of the results suggests that the significance level of all the other variables in the mediation analysis remain largely similar.

**Table 3:** Path analysis results for the relationship between the moderator effect of age on the mediating effect of HL.

	Hypothesized model			Age as moderator model		
Model parameter	β	SE	β*	β	SE	β*
CF → PF	0.115	0.012	0.385 (*p* < 0.001)	0.113	0.011	0.012 (*p* < 0.001)
PF → HL	0.101	0.022	0.200 (*p* < 0.001)	0.096	0.08	0.190 (*p* < 0.001)
PF → HRQoL	0.156	0.025	0.242 (*p* < 0.001)	0.156	0.026	0.251 (*p* < 0.001)
HL → HRQoL	0.457	0.049	0.373 (*p* < 0.001)	0.456	0.055	0.372 (*p* < 0.001)
Age*PF → HL	—	—	—	0	0.001	10.537
Fit index	Estimate			Estimate		
RMSEA	0.049			0.721		
SRMR	0.019			0.208		

β, SE, and * stands for unstandardized coefficient, standard error of estimate, and standardized coefficient, respectively.

## Discussion

This study explored the relationships among cognitive function (CF), physical function (PF) and health-related quality of life (HRQoL), and determined the mediating effect of health literacy (HL) on the relationships between CF and PF jointly and HRQoL among older adults in Hong Kong. A model was proposed and tested in the current research. Results found CF positively correlated with PF, PF associated significantly with both HL and HRQoL, supporting linkages in the hypothesized model. However, other proposed pathways were not supported -- the relationships between CF and HRQoL, and between CF and HL, were non-significant. Consequently, mediation analysis revealed HL did not mediate the effect of CF on HRQoL. Nevertheless, evidence was found for mediation of the relationship between PF and HRQoL by HL. The findings partially supported the proposed model.

Consistent with prior research, this study found a significant positive correlation between cognitive function (CF) and physical function (PF) among older adults (68–73). In previous research, the association between CF and PF existed not only among older adults with severe functional limitations (68, 69) but also for the well-functioning ones (70–73). However, the relationship became weaker after adjusting for age. This aligns with evidence suggesting that while associated, CF and PF may not have a direct causal relationship. Rather, aging itself acts as a confounding and unifying factor impacting both CF and PF over time (74, 75). Cross-sectional data limits rigorous exploration of directionality. As such, longitudinal assessment is essential to elucidate multivariate interplay between CF, PF and aging (76). Findings reinforce CF and PF as markers of aging, though additional work is needed to substantiate developmental trajectories versus direct causation.

Regarding pathways from cognitive function (CF) and physical function (PF) to health-related quality of life (HRQoL), the proposed relationship between PF and HRQoL was supported, while the link between CF and HRQoL was not. When considering their joint influence, those with higher CF showed greater PF, which in turn associated with more adequate HL. Aligning with some prior evidence (77), this positions PF as a predictor of HL, rather than simply the reverse (45–47, 78–80). Declining PF over time can reduce HL in older adults, including among those without chronic conditions, expanding on findings in disease groups (77, 80). Thus, PF impact general health knowledge access, not just functional capability. Meanwhile, the lack of direct CF-HRQoL association conflicts with studies in severe cognitive impairment (81, 82). Mild deficits likely do not affect well-being perceptions among otherwise healthy older people (83–85). That is, HRQoL links weakly to cognitive impairment itself, but stronger to resulting disability and handicap (85). As such, older adults’ undefinable cognitive changes may not relate to self-rated health or life satisfaction. Findings clarify CF-HRQoL connections may emerge only at thresholds affecting daily functioning.

As hypothesized, health literacy (HL) directly and positively associated with health-related quality of life (HRQoL) in this study. This result was consistent with prior evidence in Asian (4, 78, 86, 87) and non-Asian populations (29, 88, 89) positioning HL as impacting HRQoL appraisals. As found in Wang et al.’s study (4), HL moderates chronic disease effects on HRQoL. Conceptually, one’s understanding and application of health information would shape subjective wellbeing perceptions and evaluations (1, 28, 29, 31, 88, 89). Adequate HL enables better healthcare usage, self-management, perceived control, and calibrated expectations – all factors feeding into HRQoL judgments. Confirming this relationship provides impetus for future research on explanatory mechanisms between HL and HRQoL. Moving beyond direct links to probe intersecting correlates and cascading effects will provide a deeper understanding of how HL translates into HRQoL outcomes. Findings solidify the need to foster HL across populations as a means to engender perceptions of fulfillment and life quality on both individual and societal levels.

Critically, path analysis supported health literacy (HL) as a partial mediator (24.6% mediation) between the joint contribution of cognitive function (CF) and physical function (PF) and health-related quality of life (HRQoL). Though novel, this finding is in line with emerging evidence of HL indirect effects in related contexts. For example, a study found HL moderates chronic disease influence on HRQoL (28), corroborating the present mediating role. Additionally, recent work showed HL protected mental health and boosted quality of life during Covid-19 (29), positioning it as an intermediary facilitating factor. While analyzed with different techniques, these studies converge on the conclusion that HL enables translation of health inputs into quality of life outputs. HL mediation has also been shown between socioeconomic status and health (30), driving disparities when information utilization is inadequate. Similarly, declining body function creates difficulty that can precipitate inequities without proper health knowledge access. Findings situate HL as a leverage point through with interventions may mitigate functionality impacts on outcomes. As directly improving aging-related decline proves challenging, bolstering intermediary factors like HL may present a fruitful pathway for optimizing healthy lifespan trajectories.

Lastly, the hypothesized mediating effect of health literacy (HL) on the relationship between cognitive function (CF) and health-related quality of life (HRQoL) was not supported. This lacks alignment with some previous scholars who have proposed such a mediating role (46, 90, 91). However, since no direct association was found between CF and HRQoL in this study, examination of HL as a mediator was rendered moot. Notably, CF is often construed as integral to HL status (34, 43), yet the present findings failed to support this connection as well. Similar null findings have been reported across comparable analyses (12, 92–95), which suggest both CF and HL should differentiate between crystallized abilities (stable with age, e.g., vocabulary) versus fluid abilities (decline with age, e.g., puzzle-solving, reasoning). Specifically, only fluid CF and fluid HL may correlate significantly. In this study, HL was operationalized as a crystallized ability via the knowledge-based SF-12 components: healthcare, disease prevention, health promotion. In contrast, the multiple domains assessed by MoCA encompassed aspects of both fluid and crystallized intelligence, yet did not distinctly delineate or quantify these discrete cognitive domains. As such, the MoCA measurement could not fully capture crystallized cognitive abilities., whereas fluid abilities (e.g., attention, memory) are known to vary with hippocampal integrity (96–98). Given the SF-12’s emphasis on measuring crystallized health knowledge that is not impacted by age-related brain changes, the hypothesized mediation pathway was likely invalidated. Future studies should implement more specialized cognitive instruments to obtain a more nuanced understanding of participants’ crystallized and fluid capabilities.

The confirmed model provides new insight into reciprocal pathways between health literacy (HL) and health status. HL predicted health-related quality of life (HRQoL) as expected, but was also impacted by declining functionality. This suggests reframing HL interventions as a two-way street: bolstering HL to improve outcomes, while also supporting retention of knowledge access as health changes. Moreover, the lack of association between cognitive function (CF) and HRQoL points to additional intermediary factors needing exploration to explain this pathway. Uncovering modifiers influencing when age-related cognitive losses translate into well-being perceptions will elucidate populations requiring support. These findings highlight differential effects of CF and physical function (PF) on HRQoL to inform targeted intervention. Promoting HL throughout the aging process may optimize lifelong trajectories by both preventing declines and slowing their downstream effects. Empirical testing of continued iterative impacts between HL and changing health will clarify long-term pathways suggested by the current model. Additionally, factors like education, health status and economic status may also modify the model, with likely universal influence across variables. Research shows these factors’ links to HL (99, 100), HRQoL (101, 102), CF (103, 104) and PF (46, 105). Further exploring their impacts will allow more comprehensive analysis of the relationships examined. In summary, delineating interactive relationships advances ability to strategically foster resilient trajectories amid intersecting gains and losses. Policy must attend to modifying mechanisms on multiple pathways to facilitate aging with agency and quality of life.

## Limitations and future directions

The current study has several limitations. First of all, as a cross-sectional study, this research cannot make a conclusion regarding causality. Additional longitudinal studies are desirable to prospectively identify the predicting effect of cognitive function (CF), physical function (PF) and health literacy (HL). Second, this study was conducted in Hong Kong and the results are only based on the responses among Hong Kong older adults, therefore its application in other groups or areas is required to be further examined. Third, the current study excluded participants with impairment in CF and PF, and those with limited Chinese proficiency, thus the findings may not be applicable to the overall population of older adults. Related studies targeting on this excluded sample are required, for their health demands should be more pressing than the healthy ones. Fourth, most of the research data were collected via self-report approach, which may lead to recall bias, over- or under-reporting, or social desirability, giving in inaccuracy results. Fifth, this study did not account for baseline levels of health status, education background, economic status, and employment history, which may have impacted the variables investigated. Future research could include these potential predictors in the model to further investigate their effects. Lastly, although the current focus of this research is the mediating effect of HL on the relationship of CF and PF jointly and health-related quality of life (HRQoL), there may still be other potential mediators that have not been taken into consideration. The mechanism of HL affecting HRQoL is also essential to be explored. Future research in these areas is warranted.

## Conclusion

The current research identified a mediating role of health literacy (HL) in linking cognitive function (CF) and physical function (PF) to health-related quality of life (HRQoL) among Hong Kong older adults. The findings provided partial support for the proposed model, elucidating HL as an intermediary factor enabling translation of age-related losses into perceptions of well-being and life quality. By fostering continuing health knowledge access, downstream effects of declining functionality may be buffered. However, full mechanisms require additional investigation, particularly surrounding fluid cognitive abilities more susceptible to late-life changes. Parsing multivariate interrelationships and downstream impacts between shifting fluid capabilities, crystallized knowledge, and physical capacity will provide clarification of boundaries around the current model. Findings lay groundwork for precisely delineating how malleable factors like HL may counterbalance aging-induced losses to facilitate maintenance of self-rated health and life quality across the second half of life.

## Data availability statement

The raw data supporting the conclusions of this article will be made available by the authors, without undue reservation.

## Ethics statement

The studies involving humans were approved by the Research Committee of the University (Ref. No.: REC/19-20/0162). The studies were conducted in accordance with the local legislation and institutional requirements. The participants provided their written informed consent to participate in this study. Written informed consent was obtained from the individual(s) for the publication of any potentially identifiable images or data included in this article.

## Author contributions

BC: Project administration, Supervision, Writing – original draft, Writing – review & editing, Investigation. JJ: Data curation, Formal analysis, Methodology, Writing – review & editing, Investigation. TD: Project administration, Supervision, Writing – review & editing. HH: Project administration, Supervision, Writing – review & editing, Methodology. TK: Project administration, Supervision, Writing – review & editing. MN: Methodology, Validation, Writing – review & editing. HL: Conceptualization, Writing – original draft, Writing – review & editing.

## Glossary

BP: Bodily Pain
CF: cognitive function
GFI: the comparative fit index
GH: General Health
HL: health literacy
HLS-EU-Q47: the 47-item European Health Literacy Questionnaire
HLS-SF12: the 12-item Short-Form of the European Health Literacy Survey Questionnaire
HRQoL: health-related quality of life
MH: Mental Health
MLPE: maximum likelihood parameter estimation
MoCA: Montreal Cognitive Assessment
PF: physical functions
RE: Role Emotional
RMSEA: the root mean square error of approximation
RP: Role Physical
SEM: structural equation modeling
SF: Social Functioning
SF-12v2: the 12-item Short Form Health Survey version 2
SFT: Senior Fitness Test
SRMR: the standardized root mean residual
TLI: the Tucker-Lewis index
VT: Vitality
